# Organizational Challenges of Development and Implementation of Virtual Reality Solution for Industrial Operation

**DOI:** 10.3389/fpsyg.2021.704723

**Published:** 2021-09-22

**Authors:** Mina Saghafian, Karin Laumann, Martin Rasmussen Skogstad

**Affiliations:** Department of Psychology, Norwegian University of Science and Technology, Trondheim, Norway

**Keywords:** virtual reality technology, technology development, technology implementation, organizational innovation, managerial challenges, conservative industry

## Abstract

This research investigated the organizational challenges related to the development and implementation of virtual reality (VR) technology for operation in a conservative heavy machinery industry. The incorporation of a VR solution for heavy machinery equipment enhanced the safety and convenience of operation under dangerous work conditions. However, the development and implementation processes faced challenges. Furthermore, the adoption of the solution by users was perceived to be slower than anticipated. We aimed to explore the main challenges that the developer organization faced and how it also influenced user organizations. Due to the exploratory nature of the research, qualitative analysis was chosen, interviews were conducted, and thematic analysis was applied. The themes and subthemes were identified and discussed. The results showed the existence of challenges related to technology maturity, managerial challenges regarding communication and support coordination, workload, and multiple stakeholder management. The findings emphasize the importance of attending to the existing and potential organizational challenges before and throughout technological innovation. Theoretical and managerial implications are discussed, and a future research agenda is suggested.

## Introduction

In this paper, we investigate what is it like to develop an innovative virtual reality (VR) solution for a conservative and traditional industry. Developing new solutions using cutting edge technologies such as VR is already challenging but implementing it into an industry that values reliability and robustness above innovativeness requires extra effort to prove the technology as worthy of investment. In the present case, an innovative VR solution was developed by a team of developers in an organization that supplies heavy machines for industrial operation. The VR solution was developed to add to the safety of operators during work. The operators work in remote, challenging environments and under harsh weather conditions in forests and on the road. Operators are part of user organizations that are usually small size/family businesses (1–5 employees). Although VR solution enhanced the convenience and safety of operation, user organizations had concern about adopting a very advanced VR solution when working in remote sites, leading to slower expansion of new VR solution from the point of view of the developer organization. The developers not only faced challenges in developing an innovative VR solution, but also in implementing it in a conservative industry, including user organizations as well as other stakeholders in the same organization (an overview of all stakeholders (due to confidentiality agreements, the identities of the developers and users and the details of the patented VR technology cannot be disclosed). In this paper, we seek to identify and explore these challenges within this particular context.

Regarding the technology in question, VR applications and the use of Head Mounted Displays (HMDs) have been one of the most prominent technological trends in recent years and it is increasingly being incorporated into safety-critical industries, including heavy machinery operation. VR has been widely used in relation to safety in the form of VR serious game safety trainings for industries and emergency settings (Chalmers et al., 2009; Chittaro and Buttussi, 2015; Saghafian et al., 2020b). VR technology is a more recent phenomenon in IS (information systems) and IT innovations, which have picked up since 2016 with the release of advanced HMDs (Hall and Takahashi, 2017) such as Oculus Rift and HTC Vive. Adoption of novel technologies in safety-critical industries is challenging because system errors and failures could be costly and even detrimental, which means that more caution is practiced in these industries. Nevertheless, with the growth of the fourth industrial revolution (I4.0) and of smart manufacturing and operation, marked by more digitalization, automation, and interconnectivity, even the conservative industries need to adjust to global trends (Lewis and Naden, 2018; Saghafian et al., 2021a). According to Laudante (2017), environment, competition, and safety are the main forces for the innovation of digital technologies in I4.0. VR is an important component of the future industrial landscape (Górski, 2017) that can influence the ways of work regarding safety in I4.0. This justified the attempt of the developer team to design innovative VR solutions for their target industry to follow the global trends of technology advancement. Nevertheless, this particular technology comes with certain challenges. One of the challenges is that the time frame needed for developing and testing reliable, usable advanced technologies needs to shrink in order to keep up with the myriad of new technologies, platforms, and applications. “Robust controlled experimentation takes time, and by the time it has reached its conclusion, the capacities and capabilities afforded by technology will have changed” (Hancock and Hoffman, 2015, p. 62). Therefore, time pressure is a challenge when it comes to VR which is advancing rapidly in its technical properties and applications. In contrast to the VR technology market that is changing rapidly, the heavy machinery industry, being traditional and conservative, is slower to change than many other industries. This may be especially the case for smaller firms with more limited resources and lower risk-taking threshold. Therefore, the gap between these two industries is wider and the contrast is more nuanced than some of the other industries that are faster to change.

This brings us to the next gap that we need to address, and that is how the existing literature on technology adoption models can account for VR technology adoption and particularly in this particular context. Most of the literature about innovative technology changes, are embedded in IS literature (Eze et al., 2019) and they mostly involve changes in traditional information technology (IT) systems (Tscherning, 2012). While we have a vast body of literature about existing models of technology adoption from the IS field, VR is different because it is a more recent trend in advancement of information computer technology and is less explored in the existing technology adoption models. In addition to that, while most previous advancements in the field of IS and IT focused on 2D user interfaces and environments, VR simulates an interactive 3D environment and requires wearing a headset that can introduce ergonomic challenges and uncomfortable feelings such as nausea and dizziness that is dangerous for users in safety critical industries. Therefore, we need to know more about how users evaluate and decide to adopt VR technology compared to older technologies. Additionally, most studies about VR use focus on individual users. There is a need to understand how VR development and implementation is like when VR is often developed by one organization to be used by others and where multiple stakeholders are involved in the process. More research on how innovation within traditional firms is influenced by intra- and interorganizational dynamics and structures could contribute to the existing body of literature. The existing models and theories do not fully explore the social relations involved in new technology changes where multiple stakeholders are involved and the relations involved in a network where innovation and adoption takes place (Tscherning, 2012).

The presented challenges of developing innovative VR solution for a conservative industry, and in collaboration with multiple stakeholders, as well as the gaps in the theories in literature to account for the present case, motivated us to explore this case from an organizational point of view. This is also where the novelty of this research lies. Therefore, we aimed to answer the following research question:

*What are the challenges facing the developer organization in developing and implementing new VR solutions in their specific internal and external environments?*

In the following section, an overview of the relevant literature is provided in the theoretical framework.

## Literature Review

The literature presented in this section includes several topics that help better understand the themes developed in this research. The details on the method will be described later in the method section. However, since the themes were developed inductively, they guided the literature used to explain the relevancy of the results section. Inductive means that the themes were grounded in the data rather than suggesting that they resulted from a pure induction and it also implies that we do not claim a theoretical vacuum (Braun and Clarke, 2020). Inevitably, certain topics in the literature *a priori* informed this research, such as change management, technology adoption and VR technology. However, after analysis and inductive coding, we also uncovered ideas that aligned with other literatures that we present in this section to better communicate and relate our findings to the current body of literature, including challenges of open innovation and interorganizational complexities and coordination that were came about *a posteriori*.

In this section, a brief overview of VR technology and its relevant concepts are presented. Then the desirability of such novel technologies is explored through presenting technology adoption models that are presented next. In order to account for the complexities involved in a network of developing and launching innovative solutions, we present open innovation and interorganizational complexity theories. Finally, we present change management theories that account for new ideas, procedures, and products to better understand how the existing change management models can capture the VR technology development and implementation and address the challenges of open innovation an interorganizational complexities. This is followed by the presentation of the method for data collection and analysis, and the results of the analysis accompanied by a thematic overview of the results. At the end, a discussion of the findings, implications and conclusion are presented.

### Virtual Reality Technology

The focus of the present study is the organizational issues surrounding development of VR solution for operation. Therefore, we do not wish to provide a technical account of VR solution design and development. However, we provide a brief overview of VR definitions over time and the concepts of immersion, presence and simulator sickness. These concepts are important to consider when developing VR applications as they can influence evaluation of VR by users and task performance. Nevertheless, they are not fully understood in the literature and their relation is still not fully mapped.

VR technology is a rapidly evolving and expanding for various applications and fields, such as entertainment, military, architecture, medicine, and education (Muhanna, 2015). It has become a popular platform for training uses in safety-critical industries, a phenomenon known as VR serious game (Metello et al., 2008; Chittaro and Ranon, 2009; Chittaro and Buttussi, 2015; Gao et al., 2017; Din and Gibson, 2019), due to its benefits, such as resource efficiency, environmental friendliness, and convenience (Saghafian et al., 2020b). The literature shows that there is an increasing industrial trend toward using VR for safety at work (Damiani et al., 2018), which makes it an interesting application to explore by developers in various industries.

There are various definitions of VR technology in the literature as researchers have tried to conceptualize the term based on their respective disciplines (Muhanna, 2015). In the context of this paper, the focus is on developing VR applications that involve immersive HMDs. It has been mentioned that VR “does not yet have a single definition, but rather refers to a broad range of concepts and technologies that expand the sensory relationship with the user” (Walsh and Pawlowski, 2002, p. 299).

Virtual reality has been defined as an immersive and interactive computer-generated experience (Pimentel and Teixeira, 1993), to which the term responsive virtual world was added by Brooks (1999) to emphasize the dynamic control of users over the view. In Walsh and Pawlowski (2002), defined VR as a technology that uses multisensory channels to communicate and deliver an experience. Sherman and Craig (2003) defined VR to be an interactive medium where computer generated simulation can track users’ position and actions, replicate or enhance it to one or more sensory modalities to create a feeling of mental immersion. The terms immersion and presence are closely related to the experience of using VR.

Immersion means high involvement and engagement in what a person is doing to the extent of forgetting about the surrounding. This is form of immersion is also referred to as mental immersion (Muhanna, 2015). Physical immersion refers to being physically involved and interacting with the virtual environment. What is sensed through various sensory modalities is the result of system feedback based on the interactions. Immersion is suggested to be the quantifiable aspect of technology (Slater and Wilbur, 1995) where higher immersion results from (1) higher display resolution or vividness, (2) higher Field of View and seeing the surrounding, (3) inclusion of more sensory modalities or extensiveness, and (4) the extent to which the user of the VR system is detached from the external stimuli (Slater and Wilbur, 1995; Bystrom et al., 1999).

While immersion is the “technological quality of media,” presence is “the psychological experience of being there” (Cummings and Bailenson, 2016, p. 273) and the extent to which one feels as if they are in the computer generated environment (Bystrom et al., 1999). Wirth et al. (2007) posited that the sense of presence depends on the mental response to the sense of self orientation and the sense of perceived ability to act. Different sensory modalities, especially visual modality help create a mediated environment that creates a “place illusion” or the sense of being there (Wirth et al., 2007; Balakrishnan and Sundar, 2011 in Cummings and Bailenson, 2016).

Bystrom et al. (1999) presented a conceptual model of Immersion, Presence, Performance (IPP), based on the Slater model of presence (Slater and Wilbur, 1995; Slater et al., 1996) and the spatial fidelity model of presence by Barfield and colleagues (Barfield and Hendrix, 1995; Hendrix and Barfield, 1996a,b; Barfield et al., 1997). In the Slater model, higher presence depends on higher level of immersion, meaning the technological features of the display are important in creating a sense of presence. In addition to that the relevance of sensory modalities for performing a specific task is important and whether it is consistent with the users’ preference for which sensory modalities should be used for conveying information. In the Spatial model, presence is determined by the fidelity or similarity between spatial, auditory and haptic feedback in virtual environment and real environment, determined by technological features of VR, such as field of view, depth cues that provide depth perception or stereopsis, and motion parallax provided by tracking systems. The greater the fidelity, the greater the sense of presence. Bystrom et al. (1999) in their conceptual model postulate that better display technology features such as greater resolution, field of view, degree of freedom, tracking systems and sensor created higher immersion and better sensory fidelity because of immersion. When the VR user interacting with the virtual environment, allocates enough attentional resources to the actions and events they experience higher sense of presence and perform better (Bystrom et al., 1999). Therefore, sense of presence and the effectiveness of the VR may be linked (Lin et al., 2002).

The relationship between these concepts is not straightforward and there are many factors at play. For example, greater field of view, which is a display technology feature, was found to increase immersion and was associated with increased presence and experience of simulator sickness or motion sickness, vertigo, dizziness, disorientation, and headache among others (LaViola, 2000) up to a certain degree of field of view (Lin et al., 2002). Simulator sickness could lead to safety hazards in certain industries during job performance (Rebenitsch and Owen, 2016). The study by Lin et al. (2002) found a positive correlation between presence and simulator sickness, and a negative correlation between simulator sickness and enjoyment. Therefore, VR developers need to consider what is important for the task at hand. To what extent is immersion and presence necessary and how to create the desired level of fidelity.

Creating a realistic VR environment IS an expensive and time-consuming process (Mandal, 2013; Saghafian et al., 2020b). Cummings and Bailenson (2016) based on their review of the literature, concluded that a greater sense of presence comes with technology advancement and costly attention to design details, such as visual cues, faster update rate, wider field of view and higher tracking ability, inclusion of avatars to recreate a rich, logical and consistent environment. These authors also question if the expenditure on such details is justified, especially with a rapid technological improvement that would require constant updating. Furthermore, it is important to make a compromise between technological features and the desired outcome. This still remains a major challenge in VR technology development and its customization to target audience and task use.

In sum, there are many aspects to VR that still need to be further explored and examined regarding the specific application that it is being designed for, and what is relevant for task performance within that particular context. In addition to that the value and the motivation to use it must be greater than the costs to justify VR use. This brings us to the next topic which looks at the existing models of technology adoption and what factors contribute to decision to use or not use a new technology.

### Technology Adoption

There are various models of technology acceptance in the literature that consider the factors that are influential in the decision to use a new technology. However, we do not know to what extent the existing models can account for VR technology, and we even know less about how these models apply to conservative industries faced with the decision to VR solution. This is why we present some of the most relevant existing models in this section so that we can consider their applicability in the present context.

In this paper, technology adoption refers to the acceptance and use of the new technology by user organizations. An innovation is successful when it leads to adoption. Technological innovation is defined as “firms’ technological development of new products and new production techniques and their diffusion to other firms” (OECD, 2005, p. 10). There are many factors that influence technological innovation and adoption, namely managerial commitment, communication, training, end-user participation, and perceived justice, and work-structure factors that reflect on the extent to which the work is restructured or influenced due to technological changes (Karsh, 2004). In addition, there are two decisive factors in technology adoption: “the benefits the technology provides and the costs associated with its adoption” (Baldwin and Lin, 2002, p.1). The benefits consist of improved productivity and working conditions, and reduced energy and material use, while the costs and barriers are associated with training, software production, and maintenance (Baldwin and Lin, 2002). Barriers to technology adoption also include “difficulties in introducing important changes to the organization, management attitude and worker resistance” (Baldwin and Lin, 2002, p. 8). Other factors can be technology related, such as system flexibility, reliability and breakdown, response time, usability, and utility, which can be reflected in the technology acceptance model (TAM; Davis, 1989). While TAM is relevant for individual adopters, the organizational level models of technology adoption provide a broader view of factors that influence innovation adoption and use. In the literature, one of the most widely applied technology adoption models at the organizational level is the technology-organization-environment (TOE) model (Oliveira et al., 2014). The TOE model by Tornatzky and Fleischer (1990) has been verified with empirical evidence to support it (Oliveira et al., 2019). Because of its inclusion in the environmental context, it is deemed more complete than other organizational-level technology adoption models (Oliveira and Fraga Martins, 2011; for more detail see Baker, 2012). The environmental context is about the greater arena in which the organizations operate. Factors such as availability of technology-support providers, the decisions of the regulatory bodies, and whether the industry is generally slow or fast paced in terms of technological innovations, are amongst the environmental factors that can play a role in technology adoption. Technology implementation and adoption can be slower in conservative industries.

Conservatism refers to prioritizing stability, standardization of products and services, keeping the prices low, and being risk averse. It is in contrast to being entrepreneurial and innovative, which is marked by rapid product and service changes, flexibility, product diversification, commitment to innovation, and risk-taking (Karagozoglu and Brown, 1988; Kraus et al., 2012). Nevertheless, most industries over time will need to adjust to their environment if they want to remain competitive (Karagozoglu and Brown, 1988; Alcaide-Marzal and Tortajada-Esparza, 2007) by adjusting to their market dynamics, and technological changes (Alexiev et al., 2016; Das et al., 2018). The literature shows three environmental factors that influence the firm’s need for innovative change: environmental turbulence (rapid shifts and frequent changes in the market), market heterogeneity (differing customer preferences and technological solutions), and competitive intensity, where higher competition adds to uncertainty and risk (Alexiev et al., 2016). If the market is static, the pressure to innovate may be less, but in turbulent markets “failing to do so may directly threaten the survival of the organization” (Alexiev et al., 2016, p. 975).

In the present context, there is a rapid and competitive market that is growing for VR technology and the developers feel the need to keep up with the technology advancements in this turbulent and competitive market. When it comes to small size firms in conservative industry however, the market is more static and homogenous. Therefore, it is interesting to see how an innovative VR solution that stems from a dynamic and homogenous market can be introduced to a static and homogenous market in which both the supplier organization and the user organizations belong to. Developer team needs to introduce this VR innovation to its own organization as well as to the user organizations. Developing and deploying VR within the organization and to external organizations could be explored through the theoretical lens of open innovation literature which could provide a rich backdrop for understanding challenges that may apply to VR development.

### Open Innovation and Its Challenges

In the present case, the VR solution was developed using the VR technology and HMDs available in the market, to use and tailor it internally by developer team to make the VR solution for heavy machines with the input and feedback of users, and to implement it externally to their customers. Therefore, VR solution development and use can be explored through the concept of open innovation. This concept refers to “the use of purposive inflows and outflows of knowledge to accelerate internal innovation, and expand the markets for external use of innovation, respectively” (Chesbrough, 2006, p. 2). This implies that open innovation can include inbound innovation “sourcing and acquiring expertise from outside the organization and scanning the external environment for new information to identify, select, utilize, and internalize ideas” (Gentile-Lüdecke et al., 2020, p. 1093). Open innovation can also include outbound innovation “purposive commercialization of internally developed ideas in the organization’s external environment” (Gentile-Lüdecke et al., 2020, p. 1093). Open innovation is usually initiated within the organization’s research department to bring monetary value and is protected by intellectual property rights (Piller and West, 2014). This is consistent with the case explored in this paper.

In his work, Chesbrough (2004) highlighted that managing open innovation requires planning ahead, defining resources to be used, knowing the competitor’s capability and resources ahead of time but, in addition to that, being open to adjust and adapt planning during the process as new information is gained. However, planning ahead when there are many uncertain factors is not easy. Furthermore, the internal factors in the organization can enhance or diminish the success of open innovation (Santoro et al., 2019). In managing open innovation, a number of decisions need to be taken, including how to gain knowledge from external sources, how to manage the patent registration and protection, how to coordinate internally for carrying out open innovation, and how to determine success (Piller and West, 2014). This inherently requires balancing the advantages and disadvantages. The advantage of it is that incorporating open innovation into the organizational strategy enhances the organization’s capabilities (Chesbrough and Bogers, 2014). It enables organizations to innovate faster and cheaper and to remain competitive (Santoro et al., 2019). Moving from close innovation (innovating internally and with controlled conditions) into open innovation, however, also means less control and more technological uncertainties, challenges of working with multiple partners, and challenges related to division of tasks and responsibilities and communication, as well environmental and market uncertainties (Chesbrough, 2004).

Technological uncertainties may need more time and resources to be overcome than expected, and the organization may need to embark on its absorptive capacity to gain technological knowledge. In order to access the required hardware and software from external sources, organizations work with their partners and customers (Gassmann and Enkel, 2004; Piller and West, 2014) to “combine the outside-in process (to gain external knowledge) with the inside-out process (to bring ideas to market)” (Gassmann and Enkel, 2004, p. 12).

Challenges of working with multiple partners are manyfold. These include different cultures, managerial structures, different expectations and risks of not having the needs met, leading to loss of time and resources, and deviation from the business model and investment plans (Santoro et al., 2019). Having user participants during development process that are engaged is essential. Users often have substantial knowledge about the product that they use, but it is difficult to articulate and transfer their knowledge to the developer organizations about how it can be further improved (Ooi and Husted, 2018). This makes it difficult for developers to decide on how to work with the users’ feedback sometimes. While the developers may have the challenge of “selecting” the right feedback (Santoro et al., 2019), the managers dealing with multiple parties have the challenge of selecting the right people for the project. Working with partners requires a willingness to collaborate and to commit to delivering results on time, transparent communication about what is required, and allowing for more flexibility in collaboration (Sieg et al., 2010). If the partners are geographically dispersed, the various sites will have extra challenges collaborating across sites, may not have enough ties or may have different power dynamics at their local site. All of which makes collaboration even more complicated (Boh et al., 2007). If the parties involved are spread geographically and culturally, management needs to adjust the strategy and incentives structure accordingly to overcome cultural barriers (Santoro et al., 2019).

Challenges of task division and responsibility are about balancing the workload between daily tasks and added tasks due to the innovation that is in progress (van de Vrande et al., 2009). This can result in role ambiguity and work overload. Role ambiguity or role clarity is about not having sufficient job-related information. Work overload and role ambiguity due to ongoing changes without much feedback have been found to cause work-related stress (Zaman et al., 2013) and was mentioned to be more damaging than other stressors (Andersén, 2017). Time pressure due to work overload and role ambiguity, causing anxiety, and decrease effective performance (Murali et al., 2017). This can be more damaging to change process. Reduced engagement and reduced sense of ownership, especially when it comes to innovations, could even lead to the “not-invented-here” syndrome (Antons and Piller, 2015). This is an opposing attitude to external innovations and ideas, leading to reduced collaboration (Santoro et al., 2019). This attitude of detachment from innovation can reduce willingness to care and invest in making an invention work.

To mitigate the aforementioned challenges, management should select and clarify the appropriate goal to be achieved, by formulating the requirement or the problem clearly, and should facilitate internal and external communication, allocate sufficient time and resources, and divide roles and responsibilities (van de Vrande et al., 2009; Sieg et al., 2010; Santoro et al., 2019). Organizational culture, strategy and internal structure, the incentive system, and the general attitude toward innovation need to be adjusted (Sieg et al., 2010; Das et al., 2018; Ooi and Husted, 2018; Santoro et al., 2019). Therefore communication, coordination and collaboration are essential for directing the open innovation process toward success. This is possible when the complexities within the network of stakeholders are acknowledged and addressed.

### Interorganizational Complexities and Coordination

Inherent in open innovation process, is the involvement of multiple partners, whose collaboration needs to be somehow managed. Interorganizational collaboration is working together based on continuous communication (Majchrzak et al., 2015). Collaboration could become more challenging when industries outsource or work with other parties (Johnstone et al., 2005) which in turn blurs the organizational boundaries, a phenomenon referred to as interorganizational complexity (Milch and Laumann, 2016). Interorganizational dynamics can change over time with regard to six aspects: (1) the goal of the collaborative relationship, (2) contract (transactional and/or relational), (3) the nature of interaction between stakeholders (competitive and/or cooperative), (4) the power dynamic for decision-making, (5) the organizational structure in terms of roles and procedures, and (6) the participating stakeholders (Majchrzak et al., 2015).

To foster a functional collaborative network, three conditions must be met: (1) the technical infrastructure to facilitate information exchange, (2) organizational structure that promotes internal and cross-collaboration, and (3) organizational culture marked by openness to new possibilities and adaptability to new conditions. In addition to these conditions, the interaction between dedicated agents from each organization or party is required to set the course for further collaboration and actions (Comfort et al., 2006). One of the ways to manage interorganizational complexity is to enable simultaneous sensemaking among all stakeholders (Saghafian et al., 2020a). One facilitator that can be deployed in such situations is getting help from agile coaches. The purpose of engaging agile coaches is “to help teams find good ways of working (and keep improving them), have a sense of autonomy and ownership, be motivated, and feel like coming to work” (Bäcklander, 2019, p. 47). These agents focus on the quality of interaction between various stakeholders (Bäcklander, 2019). This is an alternative to traditional management and helps self-managing teams through adaptation and innovation in dynamic contexts. Agile coaches mainly try to support leadership by creating a balance between operational leadership, which focuses on regular routine operations, and entrepreneurial leadership, which is about being innovative and creating new knowledge and routines. This balance is referred to as enabling leadership (Uhl-Bien and Arena, 2018). The coaches engage in practices of increasing context sensitivity among stakeholders, support other managers, redirect focus on the values and principles, monitor group dynamics, highlight the areas of conflict, and encourage constructive dialog among stakeholders (for more detail, see Bäcklander, 2019).

Managing the challenges of open innovation is an important factor in innovation success of organizations (Chen et al., 2015) inherent in organizational change management. If the process is not managed well, the technology, no matter how well designed, can go obsolete. This brings us to the next topic that is important in implementation which is change management.

### Organizational Change Management

There are a number of change management theories in the literature. Some of the most famous models include the three-step (unfreeze – change – refreeze) model by Lewin (1947) (for more detail see Lewin, 1947; Cummings et al., 2016). Armenakis and Harris (2009) developed a model of change readiness, including: (1) discrepancy, (2) efficacy, (3) appropriateness, (4) principal support, and (5) personal valence (Armenakis and Harris, 2009). Cummings and Worley’s (2015) model of change includes: (1) motivating change recipients, (2) clarifying the reason and mechanism of change, (3) providing political support, (4) planning for transition, and (5) maintaining change momentum (Cummings and Worley, 2015). According to Mento et al. (2002), Kotter’s model (Kotter, 1996) is one the main change management models (for more detail, see Mento et al., 2002). The model by Kotter (1996) emphasizes what works and what does not work and is based on how people perceive change (Brisson-Banks, 2010). It introduces a model of change implementation involving eight steps: (1) create a sense of urgency, (2) create a guiding coalition, (3) create a vision and strategy, (4) communicate the change vision, (5) empower broad-based action, (6) generate short-term wins, (7) consolidate gains and further changes, and (8) anchor change into the culture (for more detail, see Kotter, 1996; Appelbaum et al., 2012). This model was found to be relevant for creating and deploying an innovative corporate culture (Gupta, 2011). It has been stated in the change management literature that “Kotter’s eight-stage process was designed for the 21st century and in the need to bring innovation sense to corporate leadership” (Gupta, 2011, p. 141). However, change is not one size fits all and it can be too complicated to be managed in steps. Nevertheless, we believe that change management literature could benefit from the contributions of this paper in accounting for challenges that arise in this particular context.

## Method

In order to decide on the most suitable method we gathered primary information about the context by talking to the upper managers of the developer organizations to get an overview of the process and parties involved. The VR solution is used for heavy machinery operations in forestry lifting. The machine that is equipped with VR solution enables the operators to conduct tasks without leaving the vehicle to an outer cabin. They can remain indoors, put on the HMD, and see the live images of the surroundings up to 240 degrees. The operators use the HMD to get the live camera image, target their object and engage with it using the controllers or the joystick. Therefore, they can conduct their task from the safety and convenience of the machine. In the older machines the outer cabin would be mounted externally on the machine. With the VR solution there is no need for the outer cabin. This means that the total weight of the machine will also be reduced, resulting in lower fuel consumption. The VR solution has been sold in 15 countries around the world. Most companies are small, 1-5 people. The number of VR equipped machines sold is about a couple of hundreds up to the present time (we do not have the information on exact number nor the clientele). The advanced technology still makes users skeptical about whether or not they want to invest in it, despite the benefits of added safety, convenience, and efficiency.

The machine is also one of the product lines of the larger organization, the supplier, that the developers work for. This supplier organization manufactures the machines in different countries and has warehouses for machine parts and equipment in different countries. The supplier organization also has technical support staff that are highly capable of fixing most issues with the machines and can fly or drive to users. The VR specific or electronic issues need to be consulted with the VR developer unit if the technical staff cannot resolve them. Independent from the technical support and VR developers that are part of the supplier organization, there are service points that offer reparation and maintenance services to the machines itself to all operators in forestry in general. They can fix and repair most of the mechanical issues. They are located in different parts of Scandinavia. The users can drive to these service points or call them for help. However, these repair shops may not have sufficient knowledge and specific expertise as the technical support staff and VR solution developer team about the VR equipped machines. They can receive training from the supplier organization if they want to. To give an example, they can be compared to body shops that can fix most cars but are not brand representatives of a specific car manufacturer. In the following section, we present how interviewees were selected, followed by the presentation of the data collection and analysis process, and the decisions made during the course of research, along with the justification of choices made. Ethical considerations are also presented.

### Sampling Strategy and Participants

The management in the developer organization recommended the primary interviewees from the development team. These interviewees further introduced the sales and support teams’ staff and end users. There were 11 participants, including nine employees from different departments of the developer organization, including research and development, sales and marketing, after-sales, and technical support, as well as two end users, each representing their own family/small organizations. These users were also involved during the development process and were providing feedback on the prototype. Therefore, they were informed users who had insight in the product. They were also pioneering in purchasing the product. They continue working with the developer team even after product launch to provide further feedback and suggestions. All the participants were male, and they ranged in age between 30 and 60 years old. Their educational background ranged from high-school level to a master’s degree at the university level. Their positions ranged from managers of departments to sales staff, as well as technicians and operators in the field. Their tenure in the respective organizations ranged from 2 to 40 years. An overview of the stakeholders involved in development and implementation, as well as support for VR solution and their geographical locations is shown in Table 1.

**TABLE 1:** Overview of the organizations involved in the development and use of the technology and their location in Europe.

Organizations involved	Location in Europe
User organization 1	Nordic country 1
User organization 2	Nordic country 1
Developer department in supplier organization	Nordic country 1
Repair shops spread out in Scandinavia	Nordic country 1
Manufacturing department in supplier organization	Central Europe country 1
Warehouse department in supplier organization	Central Europe country 1 and 2
Technical support department in supplier organization	Nordic country 2

### Data Collection

A total of 11 interviews were conducted from June 2019 to May 2020. The participants were spread across countries mostly in the Nordic region. The end users were working in remote and inaccessible areas. As part of the data collection collided with the COVID-19 pandemic, field interviews and traveling were not possible. Interviews were conducted in person and only after informed consent was obtained. In the case of a language barrier, a translator who was familiar with the industry and product from the supplier organization was called to help. It was ensured beforehand that the interviewee would agree with translator’s help and the content was verified by the main interviewee who had a basic knowledge of the language spoken and who then rephrased the text to ensure that correct information is obtained. The translator was completely impartial during the interview and only helped with language barrier on a few occasions. He was trusted by both supplier organization and by user organizations who stated that prior to interviews. The interviews with users and developers were conducted in person. The interviews duration ranged from 1 h to 90 min. The interviews with the developers were conducted in the premises of the developer organizations in a private room and one on one, as they were proficient in English. The interviews with users were conducted in the tradeshow, in a quiet location and with the help the translator who also introduced the users to us. The remaining six interviews with the other members of the supplier organization including sales team and support team, were conducted online or via telephone. All the interviews were conducted by the same researcher who has knowledge and had experience with interview techniques. For the online or phone interviews, there were no challenges of language or connection. The same general format of the interview guide was used for both in person and online interviews, adjusted based on the role of the interviewee in the development and use process.

An interview guide was prepared for our semi-structured interview. It included (open) questions about (1) the interviewees’ background information (role, seniority, and educational background), (2) workplace (typical workday and the workplace atmosphere), (3) previous experience with new technologies in their workplace and how that compared to the VR solution, (4) how they viewed the VR solution, (5) how the VR solution was introduced, (6) how the communication and feedback process was, and (7) what they thought would be the next step for this solution. We would also ask if there were anything they would like to share that we had not asked within the confidentiality agreement of their organization. While these general questions were asked of all interviewees, some specific questions were asked of developers about the development process, where the idea for VR solution came from, how was the development process in terms of challenges, how are decisions made and what was the goal of this development. The users were asked more specific questions about the adoption process, why they decided to purchase this solution, was it voluntary to use it, how often they used it and what was the training and support process like. An example of the anonymized interview guide is presented in the appendix.

Having a general interview guide with open-ended broad questions was very helpful to understand the context while giving freedom of expression to interviewees and giving them space to mention what they think is relevant and important. We then further probed to gain a better understanding of the situation based on the information that the interviewers provided. This method provided a rich account of data that then will be used to develop the themes. After probing and investigating the information provided on one topic and ensuring that the interviewee has provided all the information that he could, we would then refer to interview guide to continue the interview based on the following topic in the guide, if it has not been covered already in the information given by the interviewee. This general guide was tailored when needed. For example, when interviewing a developer, we would ask how they introduced the VR solution to the users, or how was the design and development process. When interviewing the users, we would ask how they were introduced to the VR solution and how was it implemented in the organization. However, from an organization change perspective, we would be more interested in processes and dynamics, which induced us to ask context related question about work and workplace and processes of change. This is also based on our interest, from an organizational (technology) change perspective and prior literature, for example the importance of attitude toward new technologies, management, and communication throughout the process.

The semi structured interviews with open ended question allowed for a wealth of information and data to be obtained. Interviews were then transcribed verbatim and anonymized as requested by the developer organization by the same researcher. The transcripts were transferred to NVivo 12 software for analysis.

### Data Analysis

**FIGURE 1:**
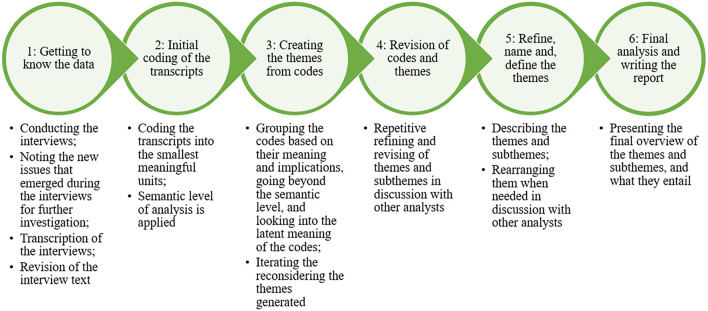
Overview of the six phases of thematic analysis.

This research was conducted within the post-positivistic paradigm, where there is a truth, but it cannot be fully observed; it is “imperfectly apprehendable” (Guba and Lincoln, 1994, p. 110). The ontological standpoint was critical realism, which aims to identify the mechanisms and structures underlying actions and events (Bisman, 2010). The data was analyzed using thematic analysis. The reason thematic analysis was chosen is because “through its theoretical freedom, thematic analysis provides a flexible and useful research tool, which can potentially provide a rich and detailed, yet complex, account of data” (Braun and Clarke, 2006, p. 78). The research was explorative and aimed to find out which issues affected technology development and use. The interview guide questions were designed in such a way that they would provide rich account of data on the context, processes and dynamics. The obtained answers were all transferred as text without interview questions into NVivo for analysis to identify the patterns and themes across the data set, using the six phases of thematic analysis including: “(1) data familiarization and writing familiarization notes; (2) systematic data coding; (3) generating initial themes from coded and collated data; (4) developing and reviewing themes; (5) refining, defining and naming themes; and (6) writing the report” (Braun and Clarke, 2020, p. 331) (see Figure 1; Saghafian et al., 2021b).

In the first phase (becoming closely familiar with the data), we transcribed the interviews verbatim and read through them thoroughly, while taking notes on initial thoughts and impressions. In the second phase, which consisted of creating the initial codes, we coded for the smallest meaningful unit of text which could convey an idea (Braun and Clarke, 2006), a notion or an issue. We noted down our ideas, impressions, and reflections to keep an audit trail to reflect on our thought processes. In the third phase, the generated codes from the text were reviewed to search for commonalities or content and they were grouped together to subthemes and themes. According to Braun and Clarke(2006, p. 82), “a theme captures something important about the data in relation to the research question and represents some level of patterned response or meaning within the data set.” In the fourth phase, the codes were iteratively reviewed and recategorized to obtain refined themes. This was done through repeated discussions with the research group to make sure that the codes and the themes were clear, distinct, and well categorized. In the fifth phase, the themes are defined and named for the last phase to produce the report on the findings. Using NVivo, meant that we first read the transcripts stored in the files, we coded for smallest meaningful units and notes what that extract was saying by giving the code a descriptive name to account for its content. This was done for all the transcripts. The codes were stored into containers in NVivo that are called nodes. These nodes were grouped based on the pattern or message or content observed, forming the subthemes and the subthemes were grouped into themes. Therefore, a hierarchical overview was created from codes at lower level into themes at upper level. This is a back-and-forth process that requires reflective and deep thinking and prolonged engagement with the data. The resulting themes are then reported and elaborated based on their content. The interviewer conducted phases 1–5 and reiterated phases 3–5. The consistency and arrangement of subthemes and themes were then discussed with another researcher, leading to adjustments in phases 3–5. This lengthy and iterative process ensured that the themes that were well-developed and represented the data in a clear and consistent manner. The report in phase 6 was written by researcher 1 and reviewed and by additional two researchers in the team who had knowledge of the case and the topic. The report was then presented to the developer organization for verification.

The analytic process was inductive, meaning that the content was coded into subthemes and themes in a bottom–up approach (Patton, 1990). Inductive implies that analysis is “grounded in’ the data, rather than ‘pure’ induction, because you cannot enter a theoretical vacuum when doing TA. Paradigmatic, epistemological and ontological assumptions inescapably inform” (Braun and Clarke, 2020, p.331). Nevertheless, we do not select a theoretical lens nor a pre-existing coding framework to code the data. Rather, in this reflexive thematic analysis we code in an open and organic way to develop themes (Braun and Clarke, 2020).

During development and rearrangement of subthemes codes to subthemes and to themes, we paid more attention to the underlying implications and applied a latent level of analysis. Latent coding “starts to identify or examine the underlying ideas, assumptions and conceptualizations and ideologies that are theorized as shaping or informing the semantic content of the data” (Braun and Clarke, 2006, p. 84). This can provide a more enriched account of what the data reflects on, the major themes, the possible relationships between the themes, and the gaps or dynamics that need further attention. For example, when one interviewee stated that “It is too few customers with [VR technology], so the repair shop staff are not trained and have no knowledge,” this could be initially coded as “lack of training due to few customers,” but on the latent level it is regarded as a problem for “external support to user organization,” which is further part of “organizational challenges in external communication and support.” The code showed how the repair shops did not invest in training because they thought there are not enough users, but it also implies that users will be discouraged from using new technologies because they do not get good support, and this could lead to fewer potential users wanting to try the new technology. Therefore, there is a vicious cycle that makes VR diffusion into industry is slowed down. In addition, this reflects on how the developer organization had difficulty communicating and coordinating the importance of training to support users across different parts of the organization that are somehow linked to this new technology. They may have difficulty convincing the repair shops that training and knowledge are essential for promoting this technology and supporting current and future potential users. This also reflects on strategic differences amongst stakeholders involved.

In conducting qualitative analysis, ensuring credibility and trustworthiness as much as possible (Nowell et al., 2017) we made sure to represent the views of all the interviewees in an unbiased way to ensure credibility. The coding was unanimously applied to all the interview data regardless of the contradictory content, and the themes reflected on all the issues mentioned. Therefore, disconfirming evidence was also taken into account (Smith, 2015). We provided a thorough description of the context based on the interview data to ensure transferability. We kept a reflexive journal to keep track of our thoughts and analysis processes. The stages of coding were documented to ensure dependability. We provided quotes from the interviews to ensure trustworthiness and auditability (Bisman, 2010). To ensure triangulation as much as possible, we interviewed people from different parts of the organization, and we verified the report with the interviewees.

During the research process, there can be a certain level of bias introduced due to the role of the researcher, which may influence the data collection and analysis. For example, the researcher has a background in organizational psychology and is interested in organizational issues while the developers are engineers or programmers, and the users are operators with technical degrees but with years of experience in generations of heavy machinery operators. Therefore, the backgrounds are widely different. Another possibility for bias could emerge in probing interviewees’ answers. This is mainly done based on what the interviewer believes to be important for understanding the context. As such, in such studies, pure objectivity cannot be claimed but it can be strived for. This is why we placed equal emphasis and attention all the data obtained from all the interviewees during the analysis.

### Ethical Considerations

Participation was voluntary, and informed consent was collected prior to the interviews. All the collected data were anonymized, and the participants’ information was stored separately from the rest of the data. Any revealing information about the identity of the organization and interviewees was kept confidential through the paper. Participants were reminded that they may not disclose any classified information regarding their job duties.

## Results

**FIGURE 2:**
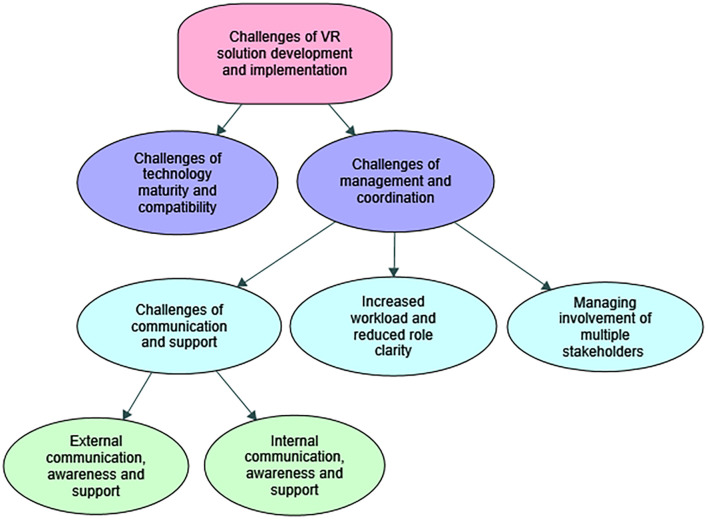
A thematic map of the challenges of the developer organization for the development and implementation of the VR solution in the specific context.

In this section, the results of the thematic analysis of the interviews are presented. We first present the detailed description of the context that was obtained based on the information acquired during interviews, followed by a thematic map of the challenges provided in Figure 2. An overview of the themes and subthemes with examples of quotes is Supplementary Table 2.

### Context

The context includes an overview of the developer organization and the technology development process for the targeted users in the heavy machinery industry. It also provides an overview of how the users or operators can ask for support and communicate about the technical issues to the developer organizations.

#### The Developer Organization, the Virtual Reality Solution, and Its Development Process

The development of the VR solution started in 2014 with the idea of increasing the operator’s safety at work by removing the operator from an outdoors cabin to indoors, such that the operator did not have to leave the machine to perform his or her tasks. Therefore, when the machine was stationary, the operator could use the controllers (also referred to as levers or the joystick) to operate from within the machine. In 2016, with the launch of Oculus Rift to the market, the idea developed such that the user would wear the HMD while inside the machine and see site of operation through images captured and sent to HMD in real-time. The operator could then target the object and interact with it using the controller or the joystick that was equipped with sensors. Hence, the concept phase started. After collaborating with several external partners and changing partners during the process, based on developing requirements and conditions, a prototype was developed. The prototype consisted of the machine that is produced by supplier organization that the developer team works for, the software and other VR component such as HMD, camera, sensors, controllers. The prototype was tested and improved into a demo model that was presented to the industry’s exhibitions. Due to confidentiality agreements and patents, no further detail about the prototype and end product can be disclosed. The demo was received with positive public response, which encouraged upper management’s approval of the production and investment with a tight deadline (see quote 1). The developer team was given 8 months to prepare the end product for the next exhibition in 2016. According to the developer organization, the competitors were investing in similar solutions; however, they were limited by patent rights are already in place concerning this particular application. Therefore, they could not replicate it and would need to modify the application. The end product was considered to be a success by developers (quote 2) and users (quote 3).

The main advantages of the VR solution, that were also mentioned by the users, were increased safety, convenience, and profitability. Not having to go outdoors in harsh winter weather conditions when surfaces can be slippery and dangerous was the main advantage of using the VR solution for operation, despite the short distance to the outdoors operation. Being able to remain indoors with air conditioning in the summer and a heater in the winter and to be protected from the harsh weather conditions enhanced working conditions (quote 4). As for profitability, the developer and user organization stated that replacing the old solution with the VR solution reduced the weight carried by the machine, reduced drag caused by the wind while driving, and consequently reduced fuel consumption. Reduced weight means that approximately an additional 400 kg of load could be carried and delivered to buyers, thus increasing profit (quote 5).

The main disadvantage of the VR solution, based on the users’ feedback, was the suboptimal image resolution due to low pixels of the Oculus Rift VR headset at the time of development. This model was replaced by newer models with better resolution. Other technical issues included a disconnection between cameras and the VR goggle, losing images, increased system sensitivity due to increased electronics and vulnerability to damage caused by harsh weather conditions. The integration of the new system into the old system made it more sensitive and susceptible to system breakdown. There were contradictory views regarding reduced system vibration when operating from outside the cabin that the users had given feedback on. While some operators enjoyed lower vibration and noise during operation, others believed that it was safer if they felt the vibration and suspension of the machine when operating it. Not feeling the movement, lifting power, and suspension were the main safety disadvantages reported. Another risk was hitting the machine with the load due to limited vision (quote 6). Developers addressed the users’ feedback by upgrading the VR headset for better resolution and improving the joysticks during the design and development process.

With regard to the internal strategies and views on the new technological solutions, it was reported that the different departments varied with respect to their view on the development of novel technologies. The developers’ department was deemed to be ambitious and mainly concerned with digitalization and not necessarily concerned about the core competence and the main product of the company (quotes 7 and 8). Therefore, there seemed to be a difference in opinion about what the strategic focus of the organization should be. However, in past years, the different departments have become more open to new technology developments, and through recruiting a younger workforce, this openness has been enhanced. Departments varied on how often they discussed new technologies and, on their views, concerning the necessity of novel solutions. The departments that were more focused on the main product line believed that the users needed to have a robust product that was reliable and not complicated, while others believed that it was a source of competitive advantage to introduce novel solutions.

#### The User Organizations, Their Work Conditions, and Their Available Support Channels

The user organizations were (approximately 70%) small family businesses with two or three machines and a few larger businesses (approximately 30%) with up to 20 machines. In this paper, only smaller organizations were interviewed. Some operators came from generations of the same line of work. Smaller family businesses may prefer the added safety for operators because of the family bonds (quote 9). Larger companies would be more concerned with replacing the operators if an accident were to happen and to attract new and younger recruits. Accidents mostly occurred during wintertime with slippery roads en route to the operation sites rather than during the operation itself. Accessibility challenges, the demanding and risky nature of operation, and the traditional line of work in the families contributed to a slow and conservative uptake of novel technologies. However, the user organization was not the only conservative party in this scenario as was explained before concerning some of the departments in the developer organization. Conservatism at the early stages of novel technologies was mentioned as being a normal reaction of users, which was observed in relation to previous new products as well. It was mentioned that it lessened over time once users saw more of the VR solutions in the industry, saw the benefits of the technology, and embraced it (quote 10). There was a support process in place where the users could go to repair shops that specialized in repairing the machines in different locations in the countries, and if the problem could not be solved, the specialists from the technical support team would provide support, and if the problem could not be solved with the VR-related solution, the developer team could provide help. However, different interviewees from different departments reported that they were the first point of contact for users. The official channels for support were usually overridden by the relationships between the users and the staff at the developer organization, based on trust and familiarity. The sales staff as a contact point in the developer team and the technical staff were all contacted by different users.

### Challenges of Virtual Reality-Solution Development and Implementation

In this section an overview of the challenges of the VR-solution development and implementation for the developer organizations is presented in a thematic map in Figure 2.

#### Challenges of Technological Maturity and Compatibility

Previous technological change implementations by the developers were mainly in the form of software updates. However, for the mounting of the VR solution on the standard product in the form of a new concept, there were many obstacles that the developers faced because of the novelty of the concept and the unpredictability of the system requirements. Designing layouts and how users view things in an immersive HMD is significantly different than traditional programming.

The VR headsets were not the highest quality in terms of resolution at the time of development, which led to lower image quality. At the beginning, the hardware that was needed to make the concept into a product was not optimal. This made decision-making difficult, and the proof of concept needed to justify the project for upper management was even more difficult (quote 11). In the meantime, the VR technology advanced and continues to advance. Therefore, there was a need for a platform to allow for easy upgrade of the software or hardware (quote 12). The pace at which VR technology advances is much faster than the heavy machinery itself. This posed a challenge with respect to the compatibility between VR technology and the very expensive heavy machines that should be able to function well for years before the user organization invests in purchasing newer models. Therefore, technological maturity at the time of development and the compatibility between different types of equipment that evolved at differing rates was a challenge for developers in this industry. The developers needed to upgrade the system without requiring the user organizations to purchase new machinery or equipment (quote 13). Other technological challenges were about data transfer and data handling capacity, where the higher data usage could cause a system breakdown. Hence, the VR-equipped machines could be affected by the integration of VR technology (quote 14). Another challenge was developing a novel product without any protocol or guide that could lead the process. This VR solution was a new idea and included many uncertainties and risks, as it would introduce a new trend in a rather conservative industry (quote 15). There could be many mistakes made, which increased the level of risk (quote 16) and the limitations and capacities of technology needed to be tested through trial and error, which would increase costs (quote 17). Hence, the developer team needed to work hard to prove that the concept was worth the investment and that the product would meet expectations.

Most of the suggestions and feedback from the test users revolved around three areas: (1) user interface, which included improving the visual quality and resolution. They also asked for one multiple functionality display on which it would be possible to navigate the menu on the VR display screen more easily without switching between menu selections too many times and to see the measurements needed during operation for precision and safety purposes. The measurements included, for example, being able to see the weight of the load lifted by the machine; (2) compatibility of the VR solution with other remote operation solutions, such as remote lock functions from another provider; and (3) ergonomic improvement, which was about the size of the controllers and their functionality.

In addition to the technological challenges, many of the challenges were embedded within the organizational structure and dynamics that were present prior to and during the development process and that were still in effect after the launch of the VR solution. These challenges are presented next.

#### Challenges of Management, Coordination, and Communication

This theme captured the challenges that the developer organization faced in coordinating the different processes and different change streams happening simultaneously that put more pressure on the employees with increased workloads, tight deadlines, time pressures, and increased uncertainty. This theme included the subthemes of (1) *increased workload and reduced role clarity*, (2) *managing involvement of multiple stakeholders*, and (3) *challenges of communication and support* that are presented next.

##### Increased workload and reduced role clarity

Certain challenges stemmed from organizational changes that occurred parallel to the development process of the VR solution (quote 18), which caused increased workload. There was a change in the structure and management of the developers’ department prior to the production of the VR solution. There was a new vision for the developers’ unit, and there was a new manager for the department. For the VR-solution launch support, there were new roles created. However, the scope of these roles and the duties over time became less specific and more diverse, causing role unclarity (quote 19). At the beginning the aim was to support product launch, but over time the role expanded to include product support, site visits to offer technical support, and becoming a link between different departments in an attempt to solve issues regarding the VR solution. Technological challenges paralleled organizational changes (quote 20). The developers, who were the early point of contact to users, often found themselves pressured by the user organization and at the same time trying to get support from other departments (quote 21 and 22) in solving user’s issues. There were reports of going beyond the initial job descriptions (see quote 23 and 24) and having to support the mechanical issues in order to provide support for the electronic issues for the VR solution, especially at the early stages of the product launch. The workload intensified with time pressures to deliver the product. After developing the prototype, the developers had a tight deadline to deliver a working product, which meant that the development process had to be rushed. The tight deadline was set by the upper management in response to the market demands and the need to stay ahead of the competition before the next exhibition. In retrospect, the developers believed that more time should have been spent on fine-tuning the products and improving the quality despite the market pressure (quotes 25 and 26).

##### Managing involvement of multiple stakeholders

Developing the new VR solution included partnerships with several internal and external parties with the required expertise (quote 27). The external partners also changed during the development process as the direction of the course of the development process and requirements evolved over time. Therefore, the challenges of negotiations with multiple partners, collaboration, and changing partnerships were added to the existing challenges and uncertainties. The machinery manufacturer was an internal partner that was geographically (central Europe) distant and culturally different from the developer team (northern Europe). They operated within a conservative industry and were perceived to be less willing to embrace the vision of the developers’ team (quote 28).

Another group of stakeholders included the potential users. One of the challenges for the developers was choosing suitable users for testing (test users), the extent of the participation, and the timing of user participation in the development process for testing. It was important that the right test users be selected. They must be experienced, loyal, and reliable with confidential matters, willing to try new technologies, willing to be critical and provide feedback, and have influence on other potential users. The developers faced a problem when one of the test users, who had the VR solution at his disposal for testing and feedback, turned out to not be using it as often and was not keen on it. This meant a loss of constructive feedback and time for developers. Furthermore, there were no inexperienced test users who could be potential targets for vocational training using the VR solution. This posed a dilemma concerning how to select test users (quote 29). It was mentioned that selection of the correct test users to minimize time loss should have been the responsibility of the management.

Regarding the timing of test users’ involvement, there were contradictory opinions. It was mentioned that test users were involved quite early in the process, but it was also mentioned that it was not early enough, and had there been earlier involvement of test users, more time and money would have been saved. This is because the developers would understand the needs of the end users earlier (quote 30). It was also mentioned that the sales team should have been involved earlier in the process, as they are more familiar with the operation and users. Not all the feedback provided by the test users was considered equally or immediately (quote 31) if it was not VR related. It was difficult for developers to work with feedback if test users did not quantify or could not break down their feedback. The safety-related concerns that were raised during the process were taken seriously and tested.

There were differing views on how the users should be involved in the development process. Some of the concerns from the developers were that the test users might not understand the vision, the potential, and the benefit of the VR solution and might not believe in it; therefore, they could not be consulted. Another concern was that if the developers and users were in interaction too much during the development process, the primary vision might get lost as the users could potentially start to steer the development process (quotes 32 and 33). Therefore, a visionary concept could become compromised. There was a comment about how sometimes the developer organization might know better than the users about what the users could benefit from (quote 34). This could be due to the different foci of users and developers. Users want more suitable machines in terms of quality and price point, while the developers are more focused on technical evolution. There is a sense of lack of immediate understanding between the stakeholders. More time and more dialog were needed to understand each other better during the development process (quote 35).

##### Challenges of communication and support

There was a difference of opinions about the strategy among different departments and whether the VR solution should be the focus of the organization. Nevertheless, it offered a competitive advantage and therefore was perceived to be important. However, collaboration between departments responsible for different parts of the VR-equipped machinery was not always smooth. This resulted in challenges in supporting user organizations when they faced technical issues. Therefore, two categories were identified in this subtheme, namely (1) *internal communication, awareness, and support* and (2) *external communication, awareness, and support*, which are presented next.

###### Internal communication, awareness, and support

This part captures the challenges for the developer organization in communicating with other parts of the organization. There was a need for more regular meetings with management and a more collaborative atmosphere within and across some of the departments when it came to supporting the end users with technical issues. Internal communication between the manufacturer and the other departments was found to be difficult (quotes 36 and 37) and much of the delay in providing support was caused by the manufacturer (quote 38). It seemed that the support givers often found themselves pressured by the users to provide an answer while they awaited an answer from the manufacturer (quotes 39 and 40). Getting spare parts from the warehouse could also come with a lead time, leading to a delayed support process, and in some cases not having enough spare parts in the warehouse itself was problematic (quotes 41 and 42). It was also mentioned that the “slow” collaboration could be due to insufficient feelings of affiliation between the manufacturer, the warehouse, and the developer organization’s support team (quote 43). Therefore, the geographical dispersion of the different units was problematic. The technicians’ teams were small and spread out, but they had regular meetings, and they did not think that the quality of collaboration suffered on account of the distance. However, there were comments about how the geographical dispersion could influence support for the user organization. It was suggested that members of various departments that dealt with the VR solution should move to the same location to work more closely together and be faster in solving potential VR-solution-related issues (quotes 44 and 45).

###### External communication, awareness, and support

The external communication challenges mainly dealt with how support was provided exclusively to the user organization for the VR solution and the challenges thereof. The support provision structure was explained previously in the context, and the challenges are explained under this section.

The upper echelons mentioned that the user organizations with the VR solution were viewed and supported in the same way as other user organizations (quote 46). This was contradicted by another comment that there was more sensitivity shown to the VR-solution users due to public opinion and market views on this new product (quote 47). Furthermore, another comment indicated that it was more challenging to provide support for the novel product (quote 48), and this coincided with downsizing the sales and support team, which was perceived as damaging to the support quality (quote 49). The staffs who are more closely in contact with the user organization believed that understanding the users and adjusting the support was not equally valued among the different parts of the organization. They mentioned that there was more emphasis on sales figures (quote 50) than on providing customized support, as user organizations had different cultures and preferences. While most of the comments were general, one comment that was about the VR solution in particular acknowledged the problems of novelty and the importance of effort toward teamwork and collective support for supporting novel products as a strategy to deal with issues arising from new technology use (quote 51).

The extent and duration of communication between the developer and user organizations was dependent on their position in the organization as well as the physical proximity to the users. It was also mentioned that in the earlier phases of the product launch, there was closer communication with the end user than later on (quote 52). Those further away from users were perceived to have less empathy and understanding of users (quotes 53 and 54). It was recommended that the developers and management personally visit the remote operation sites and see how operators work and what issues they face to gain awareness and understanding. This would establish trust, which could make users more interested in the technology. Speaking the same language was also mentioned to be important in this sense (quotes 55–58).

Another challenge was in providing accessible information to the user organizations. It was mentioned that the products are sold with a manual and that the sales staff could be contacted for any information inquiry. The technical staff could travel to the users at their operation site to provide technical support. Geographical proximity and personal contact made it easier for some of the users to get support faster than others (quotes 59 and 60). Traveling to all users could not be sustainable in the long term (quote 61). Social media was mentioned to be one platform where more information and visibility could be provided (quote 62), but one problem could be equal access for users to the social media platforms.

The users interviewed indicated that there was a lack of awareness concerning how serious downtime could be for the user organization (quotes 63, 64, and 65), and even though they could directly communicate about their problems (quote 66), they did not receive an immediate response from the developer organization. If the problem was related to the VR solution, they would need to consult VR departments (quote 67). Furthermore, the number of technical support professionals that could travel to user organizations was limited, and they might not be available to some users if they were already engaged with another user (quote 68). Users needed to be able to trust that they would get the support they need (quote 69), and they believed that the developer organization was a more reliable brand in that sense than other organizations (quote 70). This encouraged them to be loyal to the developer organization despite the challenges in receiving quick enough support.

Under the subtheme of external support to the user organization, three topics came up, including training, repair shops, user support issues, and remote support, which are presented next.

With regard to training, it was mentioned that the user organizations did not require external training from developers. They were experienced operators who preferred to practice by themselves, using VR with the real machine as opposed to simulators to adjust to the VR solution. They were aware that the simulator training did not provide the force feedback that real machines provided (quote 71). Nevertheless, the VR solution could be used in vocational schools to provide hours of training to young recruits before they operate on their own (quote 72). Therefore, it can be used for training operators. As for training, for technical issues it was found that a form of informal training for technical issues takes place as the technicians help user organizations and repair shops to solve technical issues. Therefore, they provided an informal training to the repair shops and to the users (quote 73). Nevertheless, the sales support staff believed that more training was needed for all involved in order to provide better support (quote 74).

The next topic was repair shops, which refers to places where mechanics could fix mechanical and technical issues with the machines but not so much the electronic or VR-related issues. The interviewees from the developer organization believed that despite the fact that training courses for supporting the VR solution were available for repair shops, the repair shops were not motivated to send their staff to these trainings because they believed that there were not enough users of the VR solution to justify the investment (quote 75). For better support, the managers of repair shops were expected to invest in training to support the VR solution (quote 76). The repair shop staff needed more training in detecting the source of the problem and in solving the VR-related problems. This came with more frequent exposure, as more operators embraced the new VR solution (quote 77). Also, more local repair shops were needed to provide better support and more coverage for the operators working in various remote areas (quote 78). Furthermore, the repair shops needed to be better equipped, and the users should be able to get more spare parts directly from the repair shops rather than the warehouses (quote 79). In this way, the support process would speed up, and the downtime for users would be reduced. This in turn encouraged more user organizations to invest in the VR solution. This would increase the number of users of VR solutions and would raise the demand in repair shops for the higher level of skills needed to help these users. Therefore, they mutually influence one another.

The next topic that came up regarding mitigation of challenges to providing quick support was the future possibility of more remote support. It was mentioned that remote support would be the future for the industry but, that at that time, there were problems, such as network coverage and remote viewing of the interface that the operators were seeing (quote 80). However, through another partner, the technicians had managed to provide remote support to some of the more distant user organizations (quote 81) through remote collaboration devices. They believed that this would help centralize the support system (quotes 82 and 83). The technical support staff had been very positive about the advantages of providing remote support (quote 84). It was said to be ergonomically well built and had the benefit of giving instant support to reduce downtime (quote 85).

## Discussion

This paper aimed to explore the challenges of the VR solution development and implementation process and aimed to answer the following research questions:

*What are the challenges facing the developer organization in developing and implementing new VR solutions in their specific internal and external environments?*

Interviews were conducted and a thematic analysis was applied to the data. The results showed that challenges could be grouped into technological and managerial challenges. The findings were consistent with the challenges of managing open innovation, both in terms of technological uncertainty as well as organizational and environmental challenges (Chesbrough, 2004, 2006; Chesbrough and Bogers, 2014; Alexiev et al., 2016). The case of the new VR solution in a conservative industry and with small user organizations showed that users would be willing to adopt new technologies if they were to receive timely support. This is one aspect of perceived usability and ease of use, meaning that problems can be solved, and errors can be recovered. This in turn requires that the developer organization and its multiple collaborating units are well prepared to collaborate and support each other. Therefore, the slow adoption of new technology is not always due to technological limitations or user resistance and conservatism. It is also embedded within the intra- and interorganizational structure and the coordination of vision, effort, and communication.

Within the developer organization, the organizational challenges were already present in the form of suboptimal communication across departments, the warehouse, and manufacturers. This could be due to a lack of affiliation and a common strategy and vision between departments (Boh et al., 2007; Antons and Piller, 2015; Das et al., 2018). Developing a novel product highlighted those existing dynamics. In addition to that, parallel changes, such as changes in management and downsizing of some departments, also contributed to the internal organizational issues. The resulting increased workload and role ambiguity causing stress and dissatisfaction was consistent with the literature (van de Vrande et al., 2009; Zaman et al., 2013; Andersén, 2017; Murali et al., 2017).

It was mentioned that some of the other departments that were geographically or culturally distant did not embrace the vision of the developer team, and this was problematic (Boh et al., 2007; Santoro et al., 2019). This indicates the need for more communication laterally and horizontally about the importance of innovation as one of the strategic means to survive the competition in the market (Baldwin and Lin, 2002; Sieg et al., 2010; Das et al., 2018). While the vision of the developer team was to innovate, the vision of the technical support team was to maintain reliability and robustness of the core product, the machine itself, as the key to dominating the market. Therefore, different departments become somewhat detached from each other’s vision and mission. Unifying various departments’ core competences, strategies, and visions, creating channels for easier communication and support, and enhancing logistics with the warehouse and manufacturers are other steps that would enhance future technology developments. In accordance with the literature, technology could be used for this purpose. Interorganizational complexity literature emphasizes improving the technical, organizational, and cultural infrastructure. Using technology itself could help with coordinating efforts (Comfort, 1994; Comfort et al., 2006).

This paper also shows that the presence of multiple support channels could complicate the process of maintaining an overview of technical support issues. We argue that for early-stage innovative products, it could be beneficial to have a centralized system to record technical issues and support requests. Support channels need to be further refined. There are multiple contact points for users, which is positive on the one hand, but on the other hand, it makes monitoring complaints and managing the workload of frontline staff difficult. There is a channel and an order for providing support in place, but users contact whom they know and trust and with whom they can more easily communicate. It is not clear if this will speed up support or slow it down if it is not centralized. Furthermore, with more units sold across the globe, the personal calls could become less and less effective and sustainable. With the outbreak of the COVID-19 pandemic and restraints on traveling, technicians would face problems traveling to customers and would need to use remote support even more. The remote support in real time will become more prominent. Furthermore, the future is moving toward predictive maintenance by connectivity solutions, fleet monitoring, performance monitoring, and training needs assessment.

### Theoretical Implications

In this section the theoretical implications of the results are presented and contributions to the existing literature when applicable are suggested. Further need for improvement in theoretical knowledge is discussed. The implications can be viewed in light of technology adoption and use theories, open innovation and complexity of interorganizational dynamics, as well as change management theories.

#### Theoretical Implications From Technology Adoption and Use Perspective

The findings implicate that the notion of “free of effort” put forth by technology adoption models, should be expanded to include support and fast error recovery in this context and for a complicated technology such as VR. The product was a success, as in well-liked and accepted by those who bought it, because it was efficient with respect to fuel consumption and duration of operation because one does not need to go outdoors, and it added to the safety of working conditions. This also aligned with the literature (Baldwin and Lin, 2002; Chesbrough and Bogers, 2014; Santoro et al., 2019). It is easy to use, consistent with TAM and TOE models (Davis, 1989; Tornatzky and Fleischer, 1990; Karsh, 2004; Oliveira and Fraga Martins, 2011), provided that the operators have spent time adjusting to it, but it is not easy to fix when there is a technical issue because then a failure in one component could cause the entire system to breakdown. Furthermore, while mechanical issues could be fixed at the skill level of most operators and repair shops, advanced electronics are beyond the expertise of most. Therefore, we need to revise what is meant by “free of effort” and to consider that for a technology to be accepted, perceived ease of use and usability should be expanded to cover the ease of repair and maintenance as well.

This maybe even more nuanced with an advanced technology such as VR because the knowledge and the support service for it is still limited compared to older IT and Is advances. At the same time while most issues can be resolved distantly via IT support for example, in the case of VR solution, it is more complicated. Troubleshooting is harder because the source of problem is more difficult to identify. It can be HMD, software, cameras, or vehicle itself. Therefore, while perceived usefulness of VR is consistent with the existing literature, the perceived ease of use can be more complicated. It involves more stakeholders working together to deliver support to reduce downtime. It is a matter of creating an attitude that the VR solution is easy to use despite complications that may arise.

This can be supported through insurance schemes for downtime for VR-solution users, promoting training for both operators and repair shop staff through incentive plans, and more investment in real-time remote support. These measures could help speed up the implementation and use of new VR technology in the industry. Another point that was found to be important in new technology implementation was the notion of trust. When it comes to establishing trust, end-user involvement and participation from concept to product can be a useful strategy (Dahlberg et al., 2003). Selecting the right users and their timely involvement could save money and time. There were comments that end-user involvement too early on could derail the novel technologies due to the limited vision and understanding of end users of the technology’s potential, which was consistent with literature (Ooi and Husted, 2018; Santoro et al., 2019). Literature shows that “really new-to-the-world products and radically innovative concepts are discarded because consumers fail to understand them and thus to appreciate their benefits: consumers are too conservative” (Heiskanen et al., 2007, 490). Heiskanen et al. (2007) proposed more training for users where they can gain more experience in evaluating and appreciating new technologies (Heiskanen et al., 2007).

With regard to technology acceptance theories, change needs to be introduced over time, and enough adoption time should be anticipated. The expectations of the adoption rate of innovative solutions should be based on past technology adoptions in the same industry so that it can be realistic and comparable. Besides, certain conditions need to be met for users to adopt the new technology more readily. With more exposure and visibility, and positive word of mouth between the user organizations, trust, familiarity, and satisfaction with support as part of usability criteria, technology becomes more acceptable. Users should feel confident that they can resolve the possible technical issues and develop trust in the suppliers. This is in line with the DeLone and McLean IS Success model (DeLone and McLean, 1992, 2003), which highlighted the importance of service quality as a success dimension of a new system. Service quality is concerned with the quality of the support provided by the IS and IT departments to the users and encompasses training, information, support hotlines, and a helpdesk. This dimension, therefore, deserves more attention in the technology adoption models than it has received thus far (DeLone and McLean, 1992, 2003). Nevertheless, as desirability of VR solution is more complicated than previous technologies, technology adoption models need to expand to account for novel technologies in conservative industries where the developing market and the target market are very different in their orientations toward innovation, as well as their evaluation of technology’s desirability.

#### Theoretical Implications From Open Innovation and Interorganizational Complexity Perspectives

With regard to open innovation, as mentioned by Santoro et al. (2019), the internal organizational factors impact the open-innovation success. In this case, although the product was deemed to be a success, the process of open innovation, and especially outbound open innovation, was influenced by internal organizational factors. Piller and West (2014) suggested that decisions need to be made for knowledge acquisition, patenting, and coordination. While gaining knowledge from external sources and patenting was managed well, the internal coordination between stakeholders was found to be more challenging. However, we argue that part of the coordination in open innovation could include knowledge sharing among all stakeholders rather than knowledge acquisition from external sources alone. We suggest that through coordinated knowledge sharing among stakeholders, coordination efforts will become smoother as it promotes understanding and shared vision. However, the challenges of protecting intellectual property remain an issue and need to be incorporated as an official and embedded part of the knowledge-sharing process.

Furthermore, even though the increased complexity of open innovation and the challenges thereof caused by technological and contextual factors suggested by Chesbrough (2004) can be better navigated through collaboration with partners (Gassmann and Enkel, 2004; Piller and West, 2014), different cultures, structures, and deviation from plans (Santoro et al., 2019) also pose challenges for collaboration. Therefore, we argue that a person dedicated to coordinating and managing risks and challenges could be beneficial to the process of innovation. This person could be an “agile coach” (Bäcklander, 2019).

In the case of the VR-solution development and implementation, the interorganizational complexities arose from all three sources of dynamic shift mentioned. However, it could be argued that most changes arose due to the open, innovative nature of the development, in which unforeseen challenges and adjustments needed to be made, but unlike what was proposed by Majchrzak et al. (2015), the external forces were less of an influential factor in the dynamics. The difference between the parties involved in terms of their goals, strategies, and value attribution to open innovation was the most salient force for dynamic complications (Majchrzak et al., 2015). Furthermore, the six characteristics of the interorganizational collaborative relationship mentioned in the review by Majchrzak et al. (2015) were not being actively managed by a collation, as Kotter (1996) had suggested. Therefore, more theoretical linking between organizational change management theories and interorganizational complexity theories should be made to better grasp the overlap between the two domains of theories.

#### Theoretical Implication From Change Management Perspective

Our findings make a theoretical contribution to change management theories by noting the gaps in explanations in change processes in the present context where change was initiated by one department in the organization. It was not completely top–down change because it was not initiated by the upper management and it was not completely bottom–up, because the developer department and its management are not at the “bottom” of the hierarchy. Therefore, there is a need to consider change initiated and executed by one strategic department and diffused upward, downward, and sideways in the organization. For example, Kotter (1996) highlights eight steps of change. Since the process was not completely top–down and not initiated by the upper echelon managers, some of the necessary steps were missing. For example, the importance of establishing the sense of urgency was absent until the point that the organization started to register the patent. However, this product stemmed from an idea and not the need for competition with the market. Therefore, the sense of urgency, guiding collation by change agents appointed by the upper management, development of a vision and strategy, and communication of the vision across the organization was limited to the developer team and therefore lacking in terms of active upper manager initiative. The developer team themselves established the steps to be taken through trial and error, and through showing the proof of concept in the exhibition, they tried to manifest the consolidated potential gain. This was in an attempt to convince the upper management to invest in this project. Anchoring an innovative approach in an organizational culture is an ongoing challenge, with different parts of the organization having different views on this innovative product. More theoretical work is needed to adjust the change management models of innovation when the initiative is taken by a strategic department, such as developers, who need to convince and implement change both horizontally and vertically.

Another theoretical contribution to change management theory is that when the change is not a necessity to the target industry and there is no clear end point to change implementation, many theories fail to account for change management process. In addition, the change process is more fluid when it comes to technologies such as VR. Because VR and HMDs are advancing rapidly, the VR solution also needs to be updated to keep pace with the market. Therefore, change is an ongoing process because the future of VR and operation in industry is not clear yet. This implies that there will be no refreeze stage as indicated by Lewin (1947). In the model by Cummings and Worley (2015), the last step was to maintain the change momentum. This is more applicable for a technological change that is still evolving. However, a conservative industry that should be forced to change constantly may not even be willing to embrace the idea of that particular change. Therefore, the change model should fit the target organization and their industry. Kotter’s last step of change, anchoring change into the culture, faces the same challenge. It is not clear how VR solution could change the culture of all the heavy machinery operators and whether the developers can even create a sense of urgency in the users which was the first step of Kotter’s model. Certain changes may create a sense of urgency but in this case, the VR solution is not necessarily an urgency. Therefore, change management theories should expand to account for innovative changes that are not absolutely necessary to the target industry.

In this sense, perhaps the approach of Armenakis and Harris (2009) is a more suitable approach to the present context. It suggests that highlighting discrepancy and efficacy can motivate users for accepting change and then in introduces principal support. This seems to be closer to change initiated by one department and communicated to users and upper management. They also indicate the personal valence which indicated context awareness, understanding users, empowering and motivating them. For VR technology that is evolving and is not yet regarded as necessity, this can be more suitable start point to expand on change management theories.

### Managerial Implications

Coordination and managing complexity in innovation processes can be facilitated by the inclusion of agile coaches.

In addition to that, maintaining an overview of the parallel changes in the organization and monitoring it is important. Implementing parallel changes should be coordinated in such a way that changes do not cause an increased workload for those affected. Increased communication between different parts of the organization is important for coordination. Agile coaches in different departments could facilitate intraorganizational communication and coordination across organizational silos.

Furthermore, the concept of time needs more attention, especially with regard to deciding on deadlines. While the developer organization is short on time for innovation and the production of innovative VR solutions and for patenting them to stay ahead of the market competition, consumers in the market do not feel any pressure to adjust and adopt novel technologies rapidly. The notion of time in the development process was also mentioned by Hancock and Hoffman (2015). This conflict in perceiving a time pressure could exert more pressure on the developer teams and the sales force. One the one hand, they have the pressure to perform, and on the other hand, their consumers take time to consider, evaluate, become convinced, and make a decision to try the new technology. Understanding this and trying to narrow this gap could help with perceived challenges and pressure. It could also guide the organizational communication strategy toward its target audience.

### Future Implications and Research Agenda

Future research should investigate the effect of trust between users and developer organizations on how users perceive the quality of the support that they receive. In addition, future research should explore the best ways to promote end-user training and participation in the development process and the effect of motivation and training on overcoming VR-induced motion sickness in end users. Future research should explore and test different organizational strategies for improving coordination and communication among various stakeholders. One of these strategies could be the inclusion of agile coaches and investigating how they could best function. Future research could also investigate how technology could be used to foster better communication and coordination in innovation processes. Technology could be used for better product management, for enhancing connectivity, for moving toward predictive maintenance, and for more instant communication through digital platforms. Using technology to foster the introduction and implementation of further technological change should be further explored.

Future research could also investigate the domain driven design challenges of VR solutions for industries and how complexities regarding domain can be resolved with regard to user context and involvement in development process.

In addition to these possible research trajectories, further research should be done regarding new models of change management for bottom–up innovative initiatives where the employees are the source of the change and managers and external forces are the change recipients. The power hierarchy and the enablers and barriers in this type of change could differ from top to down change models. This trajectory could be further explored.

### Limitations

More interviews would have been beneficial, but the number of user organizations available at the time of the interviews was limited, and they are very difficult to reach, as they operate in remote sites. Part of the field interviews could not take place due to travel restrictions during the COVID-19 pandemic.

Our focus in this paper has been on the challenges of developing an innovative technology for industrial operation. Therefore, we mainly focused on the developing organization and stakeholders involved in development and the technical support and sales staff. More user organizations would be desirable for gaining more insight into the target industry. However, we had limited access to this group.

The success of the VR solution was concluded based on the fact that the added VR solution worked. It gained attention in the tradeshow which in return encouraged the upper management to invest further in it. Furthermore, the two user organizations that were interviewed stated that they quite liked the technology itself. We were not granted access to sales record or market information to establish product success in other ways. We were informed by the sales department that also larger firms were purchasing the product. This limited information about how many organizations were introduced to the new VR solution and how many accepted or rejected the new solution. The user organizations that were targeted in this paper were small businesses. The operator that uses the technology also has power/influence on purchasing decision. Furthermore, we were quite limited in access to users. They work in remote areas. Cell phone signals are not always available even. The tradeshow was our rare chance to meet the operators that use VR solution. Therefore, we were limited by the attendance of the users at the tradeshow where it is possible to meet and speak to users. The developer representative in the tradeshow helped identify and contact the users. Otherwise, they were not identifiable. Nevertheless, the developer representative with substantial institutional knowledge assured us that the users interviewed were typical users that purchased and used the VR solution, with excellent operating skills and power to decide to use or to purchase the new VR technology.

We were planning to approach another industrial user for the same solution but in a different field of operation. This was an industrial plant that used the same solution on a different piece of machinery and their increased productivity and production was quite visible and recorded. This proved that the VR solution in and of itself is a success, but the context can play a role. We could not however continue the research at the industrial plant due to COVID-19 pandemic and closing of borders. We plan to do this after restrictions are lifted.

However, we attempted to interview people from different departments in different countries and from the front line to managers. Furthermore, much of the information was kept confidential by the developer organization, such as pricing and sales figures in different areas. The interviewees were selected firstly by the management and then through snowball effect, and none of the members of the manufacturers and the warehouse could be reached. Therefore, we acknowledge that there may be some level of bias in the present paper. Nevertheless, as for the rigor of the study, we attempted to establish credibility through prolonged engagement in data collection and triangulation among departments and online resources. A thick description of the context was provided to ensure transferability. An audit trail and reflexivity were ensured through keeping a research journal throughout the research in order to be aware of our position in that regard. Dependability was aimed for via iterative analysis of the codes and themes and repetitive member checking by other researchers in the team. Confirmability was aimed for by trying to reflect the various and sometimes contradictory data to ensure neutrality in presenting the evidence.

## Conclusion

The development and adoption of new VR solutions in the heavy machinery industry and in small user organizations, poses several technological and organizational challenges for developers. The organizational structure and dynamics need to be evaluated prior to new technology development in order to reduce the number of organizational challenges and parallel changes for developers. This will help developers deal with technological challenges and improve the usability of the VR solution. Improving usability, improving support functions for users, providing training and learning programs for users and support staff, and a realistic communication of anticipated adoption time based on the present context can ensure a smoother development process and facilitate the adoption of a VR solution by conservative user organizations. Involving users from the beginning of the development process can help with many technological challenges, enhance trust and communication, and encourage other user organizations to embrace VR-solution technology.

## Data Availability Statement

The datasets presented in this article are not readily available because confidentiality agreements. Requests to access the datasets should be directed to the corresponding author.

## Ethics Statement

The studies involving human participants were reviewed and approved by Norwegian Centre of Research Data. The patients/participants provided their written informed consent to participate in this study.

## Author Contributions

MS was responsible for the planning and designing the study, data collection, interviewing and transcribing, data analysis, and writing. KL was responsible for the provision of funding, planning, and the design of the study, and for providing feedback during all phases, including writing. MRS reviewed and provided feedback during the writing phase. All authors contributed to the article and approved the submitted version.

## Conflict of Interest

The authors declare that the research was conducted in the absence of any commercial or financial relationships that could be construed as a potential conflict of interest.

## Publisher’s Note

All claims expressed in this article are solely those of the authors and do not necessarily represent those of their affiliated organizations, or those of the publisher, the editors and the reviewers. Any product that may be evaluated in this article, or claim that may be made by its manufacturer, is not guaranteed or endorsed by the publisher.
