# Supplementary material for: Organizational Challenges of Development and Implementation of Virtual Reality Solution for Industrial Operation
Source: Front Psychol. 2021 Sep 22;12:704723. doi: 10.3389/fpsyg.2021.704723 (PMC8492956; doi:10.3389/fpsyg.2021.704723)
Supplement: Supplementary file 1 [file Table_1.docx]

**Supplementary Table 1**

*Overview of the quotes per theme and subtheme*

| Theme | Subtheme | Example of quotes |
| --- | --- | --- |
| The developer organization, the VR solution, and its development process |  | 1. It was actually when the manager […] was visiting at the (trade fair) in August 2014. He tested it and said we should do this, and we also got the marketing manager who also […] put in some money. So, you have quite high upper management sort of saying yes to this. 2. It’s the customer’s choice, and the customers really love it, and so every customer who has bought it is really happy with it. 3. It is fantastic, this VR. 4. In the winter (outdoors), it is -25 degrees, not so nice, and it is also raining, lots of rain and wind, but indoors, you are very safe from the cold and the rain and the wind and everything. So, it’s a big difference. 5. We can take about approximately 400 kg more load, and that is a big thing. Also, about 1% more money for every load. 6. We hit ourselves by accident because we are away and we are not there where we have our eyes (referring to sitting indoors) so that was something we tested, and we realized this was a real risk. 7. When it comes to these departments with digitalization, they are again too far and kind of reaching for skies. 8. They (developers) kind of forget about the product […] they just want to send things to the cloud […] which is not really even connected to our core competence. |
| The user organizations, their work conditions, and their available support channels |  | 1. For small companies, it is more important to secure that their family knows they are coming back home, so safety and security so that it is easy to (operate). 2. But it’s not a quick fix. You cannot sell 100 VR systems next year. It takes time […] because they are very conservative, very sensitive people, they are not really 100% sure that it is a good product but […] in about 5 or 6 years then we have another situation. So, it takes its time […] the more we sell, the more you will see (VR solutions). |
| Challenges of technology maturity and compatibility |  | 1. If you make a concept that you come up with, many will say ok this is good technology, but the cost to do it may be too high right now or we don’t have may be the hardware yet. 2. We try to be clever and create platforms that we could just upgrade easily. 3. It is more about how to make sure that the machines are expected to be there 5 to 10 years. That is a very long time when it comes to these VR applications. They are more like one year, and then there is already another version. 4. In reality you might have a different provider of goggles and then how to kind of communicate and how to make data transfer…now we use internet cables, but what if we need to send more data...I don’t even know, but we just need to believe that we can follow and keep our eyes open and be as open and flexible as possible. 5. with the environment we work in, then maybe you would probably let it be for a while. 6. It was a trend change. 7. We had no help, so we made in a way many mistakes, and we did not realize some things now to think back it would be different. |
| Challenges of management and coordination | Increased workload and reduced role clarity | 1. I think it is a new organization, new product, new technology and that mix is a lot of new stuff so that it usually creates a problem. 2. Now I have a new manager, and he has some ideas what we should do but I don’t really remember anymore what my purpose was, why did they hire me, I don’t really know because nobody knows anymore. 3. The challenges for the organization are of course the same. And again, it in a way it comes down to ownership and understanding customer and industry requirements and needs, to be honest. 4. I would say that lots of these issues and problems I solve by myself, so that is very sad, but that is reality. 5. I think it’s very important that we have the possibility to have this help from these different departments because we are on our own and we have lost of work for sales and support. 6. But the way to this point has been a lot of things that I was perhaps not meant to do. 7. So, my situation is developing to perhaps to something else than perhaps was meant 8. With that kind of stuff that is so new, you have to prove it a number of times to get the attention from the upper management to invest in it. 9. To now think back, we should have spent a couple of more months for fine-tuning things before its release, but I understand we had to come up with something because the more time we spend, the more time we give to our competition. |
|  | Managing involvement of multiple stakeholders | 1. But then you have all the stakeholders with you and then from R&D, sales, marketing, sourcing, production everything after-sales, so that’s much bigger product than before […] what we do is a combination and software. 2. They (manufacturer) perhaps are not that fast to change things. 3. Either we had to take someone directly from school, but how can we evaluate a person with no driving experience if he or she says it is not good or good. So maybe it shows that we are old-fashioned. We wanted to play safe and keep customers informed about what we are doing, and our professionals give us feedback if they see a future with the system. 4. They can do the thing right from the beginning, and it saves us money. 5. As soon as we had something, they could test on…asked for their feedback on it. And yeah, we got feedback, but it wasn’t the feedback maybe that we expected. It was other things that they were focusing on, the joystick is too big was one feedback really before we launched it and the resolution, but we couldn’t really do anything about that and those kinds of things that we knew and expected to be a problem were not a problem. 6. Some designers are quite distant from the customers, but at the same time, we don’t want the designers to go directly to the customers because they start to come up with this and that, and the customer starts to steer the development. 7. So, being a designer myself, it is frustrating when you don’t meet the customer, but at the same time, we want to use the R&D guys to develop what they are good at. Sometimes it is good that they are not directly meeting the customers. That is just the bottom line. 8. We need to try to have this bigger picture of what they are struggling and what job they want to get done, and so on 9. It takes a little while to have them (developers) work this way or else the customer doesn’t want it, and so it took a little while to get the team understand what the customer wanted because they have never done it. |
| Challenges of communication and support | Internal communication, awareness, and support | 1. The biggest problem we have when we talk about communication is with the factory. 2. I don’t know because normally when I call the customer or when I set up meeting with customer or dealer, I hope that they answer me because if they call me, I answer back, but I cannot say why they don’t answer from the factory. I don’t know. 3. The most problems we have are with the factory. 4. The customer has a problem, and he really wants to solve the problem because he is disappointed, and he calls me again and asks how it goes, and I don’t hear anything from you, and I don’t have an answer because I don’t have an answer from the factory, and so, that’s a big problem that we have there. 5. I would say that that’s the problem because when we speak with some of the departments, they never come back with answers, so you must call them back, and you must email them, and you call again, and that’s the problem. 6. It’s another story if they require a spare part; it has a lead time, and we may not be able to help in some cases, but otherwise we try to be there for the customer. 7. The challenge is that as a company we are not allowed to have big stocks, and if there is bad luck, it might take some time to get the spare parts if it is a very rare part, so it is more like this classical problem. 8. You know we have fought a lot in the two years’ time, but today we have a product that is in quite good shape, looking to how it looked two years ago. Well it’s not correct to say that they (manufacturer) do nothing, but it is a little bit hard to work with those guys. Because we are smashing a little bit from the side. We are not really part of their organization. We are a bit on the side, so perhaps not really accepted. 9. My suggestion a couple of weeks ago was to start a VR-solution department […] so that we can quickly solve problems that customers have. 10. I have already proposed a new thing to support the customer faster, and my suggestion here is that we must start a VR-solution department because now we have different people in our company who work with VR solution […] but they are not a good team because they are not working together. 11. A customer using a conventional control system is equally important as a customer using VR solution. So, there is no difference there in response from the surrounding organizations. 12. We take care of HV technology a bit more because it is more sensitive to public opinions at the moment. 13. How do you support these kinds of new products because it is quite different? If someone calls and says I don’t have a good view, what do I do now? It is harder to support that because it is new. You don’t have the statistics to prove that because initially you don’t know where the problem is. 14. There were four people, and they are two people to save money. They are not working so well just now. I hope they do something about it. 15. There are some managers […] who are more interested in sales and then get money for the sales. 16. This is such a new technology, and I think there have been internal problems dealing with these sorts of new complex products. That is for sure because you have some support staff that can deal with more mechanical things and electronics, but when it comes to computers, then they are lost, and then you have the connectivity. So, the more advanced. We think like a team to connect with service support staff that can help with computer-related issues. |
|  | External communication, awareness and support | 1. Not so much nowadays because we are done with the product. But those times we did quite often. At least someone in the group got feedback as fast as possible. 2. When you climb in the structure of the company, you drown in another things. It (communication) stops in a certain level. You need to take it further. 3. These people do not understand that we work with this product. If it does not work, we will have to wait two or three days. 4. Quite soon in the service organization or the organization you are talking to, quite soon they don’t understand where their pay comes from. Their pay comes from our customers, and they should support the customer. Quite fast you lose the customer. You just work in the company. You don’t see the customer. The real customer. Perhaps you see some other customers inside the company, but you don’t see the real customer whose machine is standing still, and he needs it to work now. Not tomorrow, not next week. He needs it now. 5. Because they don’t work with system, they have not experienced it, and they don’t see easily how it is to work with the system and the customers in the (operation) site. 6. You must know the customer. Their family and their life. That is very important, and they get interested. 7. You get a whole other thing. It is a very different culture in the south and north of the country. In the north, they really let you come home and go to their table and drink coffee. It is more important in north. In the south, they just go to repair shops. There is a cultural difference. There is a big difference in the south, middle, and north of the country. Should you work in the north, it is good to know the culture, and in the north, they like when you can speak their language, they really like it. It’s a big difference. 8. It’s not maybe so easy to find us …there are customers that we know very well, but if there is a random guy trying to ask something from us, it may be difficult to find us. 9. We are very well hidden in parent.org. We are quite distant. On the one hand, we are very close to some customers, and we are sometimes very distant, and it is hard to find technical information. 10. The challenge is that if we start let’s say when we talk about years in front, then we might be tens or hundreds or thousands of customers, and we cannot just fly people around. 11. It is hard to find technical information. If you go to the networks of our competitors, then you do just a couple of clicks to find some info, but for us you need to know us already. So, we are distant sometimes. 12. They (operators) often need to find solutions for the problems by themselves to be able to run the system because the way for support from the company’s side is too long, so they can’t wait several days for a small part to get it running because the machine needs to run. Always. You need up time. 13. These people (developer organization and manufacturer) do not understand that we work with this product. If it does not work, we will have to wait two or three days. They don’t understand we must drive this machine. 14. Something what is not so good now is that when the customer calls to order things, it takes a long time for it to come, and they (developer organization and manufacturer) don’t always answer the phone. 15. I have direct number to everyone so I can call them. 16. It is new technology. You can’t expect that every repair shop has the knowledge and that kind of problematic things, but anyhow to be successful you need to support the customer you sell the product to. 17. The slowness of response is maybe because we have a dedicated person or the specialist might be on holiday or traveling. 18. Maybe some issue with trust in the developer organization. Some of them were a little bit skeptical how they get support if they have any problems, but there aren’t any problems now. 19. They (user organizations) think that it (developer organization) is better than other companies (with giving support) so it works very well compared to other customer complaints, but it can be better, I think. 20. Because you need the force feedback. If you are running the simulator, you don’t feel it. When you are running the machine, you are going like this, you are shaking back and forth, that gives you some kind of feedback on what is happening. 21. You have the system, and you can learn […] so when you come out in the real world, you have saved many practical hours. So, it’s a very good system. 22. But we have a really good manual, and of course we are helping and training these repair shops how to solve the problem, so we are at the same time training the customer how to use it. 23. We should look into training everyone and improve support and those could be very helpful. 24. Our service points, or our dealers, or repair shops, they are not so interested in sending guys for a VR-solution course. Maybe there are not too many machines with VR solutions so they don't see the point of giving their service people any education in it because there's not so many hours to work with it. 25. You need to have the confidence in the local repair shop to do the investment in technology because it's a shift in technology for the user. 26. When you hit the repair shop with a system full of saltwater, it is like puff, nothing more. It is completely black. It is nothing to go. Then it is quite demanding to begin to find fault with it […]How do you repair something you have never seen and you don’t have the slightest idea or clue how it works? 27. (Support) is better in the south than in the north of the country because there are not so many repair shops. 28. (Better support needs) more repair shops that can help and have spare parts home at the repair shops so they (user organizations) can buy them. 29. We have such a bad coverage on the site. It is bad for phone connections. 30. We have created this kind of remote support. When the issue is software related or VR related, we are able to advise the customer to plug into the computer so that we see what they have and then connect through internet, and then we can have a remote connection and then check the system to see if the VR system is ok. 31. We want to do it (support) kind of centrally somehow, and that is why we have this remote support. 32. After that actually, I’m using also at work VR for supporting customers with their problems. So […] you have a helmet and a camera on the helmet, so I see what the technician in the field is doing, and I can actually see through his own eyes, and it has a really small display on the right eye, and he can for example show him some probing. So like an extended reality. 33. Yes it has potential […] what helps is to have a service person who has HoloLens or nice small goggles that you see through, and you get some sort of support or service that does this, or this is the moment you need to use or change some part. 34. Our customers are around the world […]. So it could be emailing and even a small problem can take days because when you are asking something and there is a time difference, it takes a lot of time with the time difference. This is really easy to connect to them, and I can see the same thing as the technician there, and in 10 minutes it is done, and the customer is happy and goes back to work. |
