# Supplementary material for: Organizational Challenges of Development and Implementation of Virtual Reality Solution for Industrial Operation
Source: Front Psychol. 2021 Sep 22;12:704723. doi: 10.3389/fpsyg.2021.704723 (PMC8492956; doi:10.3389/fpsyg.2021.704723)
Supplement: Supplementary file 2 [file Table_2.DOCX]

### **Template Developer organization, Developers:**

**Background information**

1. What is your position?
2. What is your educational background (shortly)?
3. Can you tell me shortly where you are in the organization?
4. How long have you been working at Developer organization and how long in this position?

**Work and Workplace**

1. Can you describe a typical workday for you? Only if it can be disclosed.
   - What do you do?
2. How is the social environment at your workplace?
   - What is your team like?

**Experience with new technological developments at Developer organization**

1. Can you tell me about how you develop new technologies at Developer organization?
   - Who makes the decision to develop new technologies?
   - Who is involved?
   - What is the process for developing new technologies like?
   - How often do you develop new technologies?
   - Where do the ideas come from?

**View on VR solution**

1. Can you tell me about VR solution?
   - Where did the idea of developing VR solution come from?
   - What do you think about it?
   - Who decided on developing this technology? Why?
   - What does it do?
   - What was the goal of developing it?
   - Did you know beforehand what was the end product or was it by trial?
2. What analyses and evaluation did you do on VR solution before developing the end product?
3. Did you talk to the clients about it directly?
4. Did you talk to any of the operators about it during development?
   - When did you talk about it?
   - How was the interaction between you and clients?
   - How did the client affect the design and development?
   - Did they provide feedback?
     - What did they say?
     - How did you handle it?
5. What are the most common challenges of operators working in the industry?
6. How does VR solution help with these challenges?
7. How is the VR solution different from the traditional way of operation?
8. Did it do what it was designed to do?
9. Have you ever operated the VR solution yourself?
10. Why did you think Virtual Reality technology was a good choice?
    - What were the alternatives?
    - How are the advantages and disadvantages of VR solution over alternatives?
    - What were the discussion for and against using VR solution?
    - What is the current competition investing on?
11. How was the development of VR solution different to other developments for you?
12. Is there something that you would do differently about it?

#### **Immersive Visual Technology**

1. Have you ever used immersive technology (VR, AR, other) for other projects?
   - What do you think about it?
2. What do you think about the future of VR in your industry?
   - How do you think this new technology would change how operators work?
   - How do you think the use of VR would make operation safer and more efficient?
   - Do you think that Developer organization should invest in another technology other than immersive visual technology? Why/why not
3. Do you think immersive visual technology would cause stress?
   - Did you receive complaints about motion sickness and nausea?

**Introducing the VR solution to the clients**

- - How was VR solution put into use?
  - Did you design any training for the VR solution?
    - How was the training set up?
    - Was it enough?
  - How did you provide technical support to the end users?
  - What were the most common questions and problems about VR solution?
    - How did you resolve the problems?

**Communication and Feedback**

1. How do you spread information about new technological developments?
   - Is it how you usually communicate?
   - How do you usually communication to your clients about new developments?

**Future**

1. What do you think is the next step for VR solution?

### **Template user organization, operators: technology implementation**

**Background information**

1. What is your position?
2. What is your educational background (shortly)?
3. Can you tell me shortly where you are in the organization?
4. How long have you been working at your organization and how long in this position?

**Work and Workplace**

1. Can you describe a typical workday for you? Only if it can be disclosed.
   - What do you do?
2. How is the social environment at your workplace?
   - What is your team like?
   - Do you discuss new technology with your colleagues?

**View on VR solution**

1. Can you tell me about VR solution?
   - What do you think about it?
   - What does it do?
2. What is the purpose of VR solution?
   - What were the alternatives?
   - How are they better or worse than VR solution?
   - What does do others use?
   - Why do you think your company bought it?
   - How often do you use it?
   - Can you decide if you want to use it or not?
3. How is the VR solution different from the traditional way of operation?
4. What are the most common challenges of operators working in your industry?
5. How did VR solution help you do your job?
6. Do you think VR solution was successful?

#### **Immersive Visual Technology**

1. Had you ever used immersive technology (VR, AR, other) before the VR solution?
   - What do you think about it?
2. What do you think about the future of VR in your industry?
   - How do you think VR can change how you work?
   - Do you think your organization should invest in another technology other than immersive visual technology? Why/why not
3. Did you have stress when using VR solution?
4. Did you feel nauseous when using VR solution? If so, when?

**Introducing the VR solution to the operators**

1. How did your company introduce VR solution to you?
   - Did you get training for using it? How was the training set up?
   - Did you have technical support? How was the support?
2. Did you ever say your opinion to the developers of VR solution?
   - How did they react?
   - What was the result?

**Communication and Feedback**

1. How do you hear about new technological developments in your organization?
   - Is it how you usually communicate?
   - How do you usually give feedback to your supervisors?
     - What do they usually do about it?

**Future**

1. What do you think is the next step for VR solution?
